# GDM-complicated pregnancies: focus on adipokines

**DOI:** 10.1007/s11033-021-06785-0

**Published:** 2021-10-15

**Authors:** Marta Mallardo, Sara Ferraro, Aurora Daniele, Ersilia Nigro

**Affiliations:** 1 Dipartimento di Scienze e Tecnologie Ambientali Biologiche Farmaceutiche, Università degli Studi della Campania “Luigi Vanvitelli”, Via G. Vivaldi 42, 81100 Caserta, Italy; 2 CEINGE-Biotecnologie Avanzate S.c.a r.l., Via G. Salvatore 486, 80145 Naples, Italy; 3 Dipartimento di Sanità Pubblica, Università degli Studi di Napoli “Federico II”, Via Pansini 5, 80145 Naples, Italy; 4 Dipartimento di Medicina Molecolare e Biotecnologie Mediche, Università degli Studi di Naples “Federico II”, Naples, Italy; 5 CEINGE-Biotecnologie Avanzate, Via Gaetano Salvatore, 486, 80145 Naples, Italy

**Keywords:** Gestational diabetes mellitus, Pregnancy, Adipose tissue, Adipokines, Adiponectin, Leptin

## Abstract

Gestational diabetes mellitus (GDM) is a serious complication of pregnancy and is defined as a state of glucose intolerance that is first diagnosed and arises during gestation. Although the pathophysiology of GDM has not yet been thoroughly clarified, insulin resistance and pancreatic β-cell dysfunction are considered critical components of its etiopathogenesis. To sustain fetus growth and guarantee mother health, many significant changes in maternal metabolism are required in normal and high-risk pregnancy accompanied by potential complications. Adipokines, adipose tissue-derived hormones, are proteins with pleiotropic functions including a strong metabolic influence in physiological conditions and during pregnancy too. A growing number of studies suggest that various adipokines including adiponectin, leptin, visfatin, resistin and tumor necrosis factor α (TNF-α) are dysregulated in GDM and might have pathological significance and a prognostic value in this pregnancy disorder. In this review, we will focus on the current knowledge on the role that the aforementioned adipokines play in the development and progression of GDM.

## Introduction

Pregnancy is characterized by complex endocrine and metabolic adaptations aimed at satisfying the increased energy demands necessary for the development of the fetus [1]. One of the most important metabolic changes in pregnancy regards the production of insulin, hormone that promotes maternal nutrient storage to support the energy demands of the fetus [2]. In the later stages of pregnancy, a state of insulin resistance, promoted by the action of several hormones [2, 3], determines an increase in blood glucose that supports fetal growth thought the transport across the placenta; although glucose levels rise, the greater secretion of insulin ensures the maintenance of a normoglycemic state in the majority of women [2, 3]. However, when β-cells are unable to compensate for insulin resistance, gestational diabetes mellitus (GDM) usually initiates [4]. GDM is a common pregnancy complication associated to short- and long-term healthy problems for both the mother and the fetus including birth complication and type 2 diabetes mellitus, T2DM [5]. Several risk factors have been identified in the development of GDM, including family history of GDM or T2DM, insulin resistance, advanced maternal age, smoking and obesity [6, 7]. Several organs including the brain, liver, muscles, and placenta participate and cooperate in the development and progression of GDM through hormonal regulation [8]. A crucial role in the pathogenesis of GDM seems to be also played by adipose tissue (AT). Indeed, AT is considered an endocrine organ that, through the production of adipokines, regulates many biological functions and influences pregnancy as well as several pregnancy complications such as GDM [9].

The aim of this review is to provide an overview of the main adipokines secreted by AT that may possibly participate to GDM development.

## Adipose tissue role in the pathogenesis of GDM

**Fig. 1:**
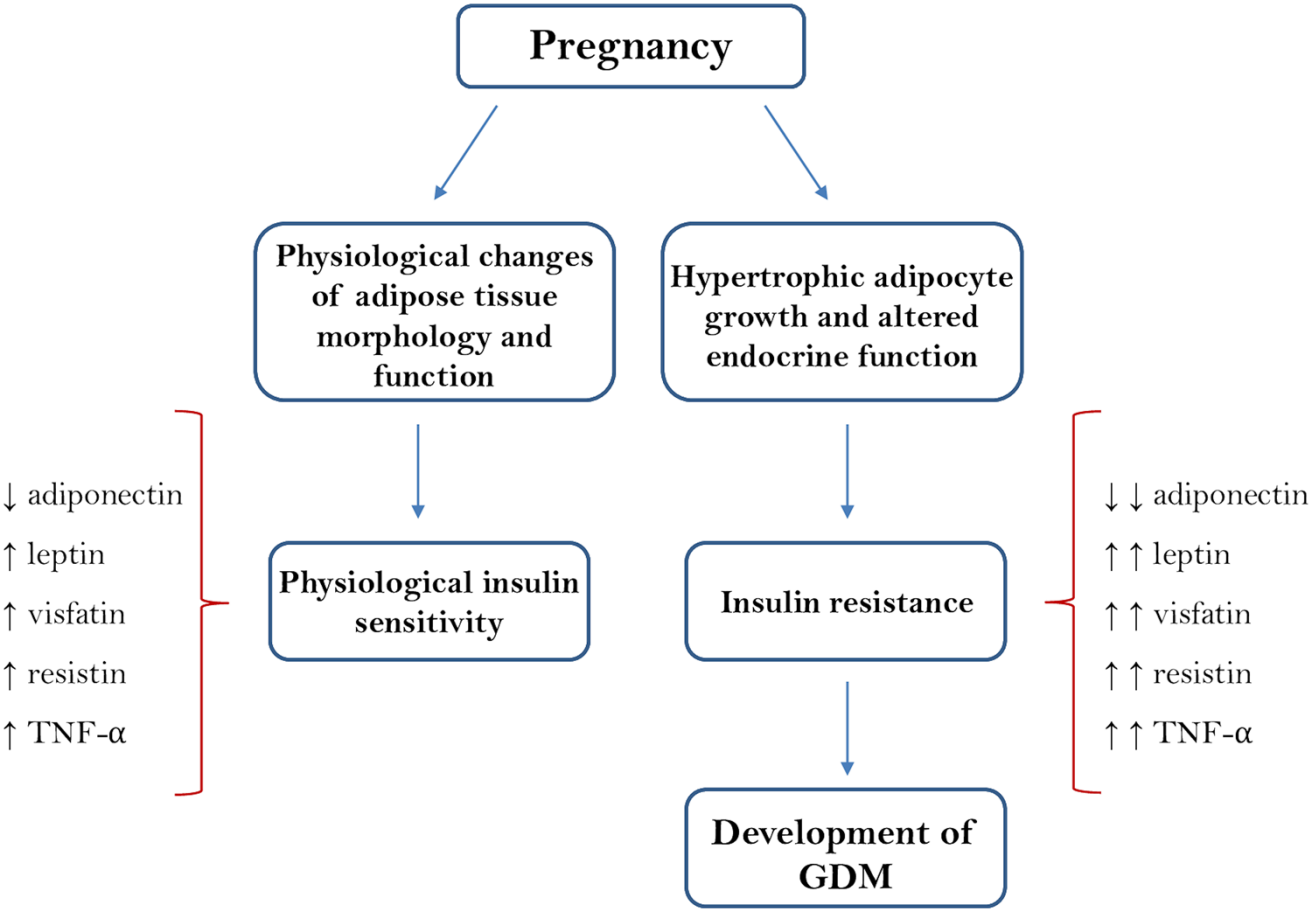
A simplified scheme of maternal circulating levels of the major adipokines involved in physiological and GDM pregnancies. Several adipokines, including adiponectin, leptin, TNF-α, resistin and visfatin, are involved in the regulation of maternal metabolism and gestational insulin resistance. Insulin resistance associated with physiological pregnancy is further improved in GDM

Beyond its role of energy storage, AT represents an important endocrine organ that regulates many biological functions through the production of hormones, known as adipokines [9]. The endocrine function of AT appears to influence not only the physiological pregnancies but also pregnancy complications such as GDM. During a physiological pregnancy, there is an increase in the AT mass functional to the mobilization of fat to sustain the fetus growth [10]. Several studies reported that GDM is associated with hypertrophic adipocyte growth together with a downregulated gene expression of insulin signaling regulators [11]. In fact, hypertrophic growth of adipocytes can compromise the functionality of AT causing a dysregulated production of adipokines [12] which, in turn contributes to impare insulin signaling inhibiting the release of insulin from β-cells and promoting the onset of GDM (Fig. 1) [13].

Adipokines are a family of proteins synthesized and secreted by AT [14]. These adipocyte-derived hormones take part in several metabolic functions; some of these are mainly involved in the immune responses and in the modulation of the inflammatory processes, while others primarily regulate glucose and lipid metabolism [15]. Despite the main site of adipokines production is the AT, during pregnancy, placenta secretes several adipokines such as adiponectin, leptin, resistin and visfatin which seem to be involved in the regulation of maternal metabolism in normal and pathological pregnancy conditions [16]. Adipokines play a key role in numerous physiological processes (regulation of energy consumption, inflammation, modulation of the immune response, reproduction, and angiogenesis) that influence the outcome of pregnancy and fetal growth, thus representing important factors in the pathogenesis of GDM [15]. Importantly, altered levels of adipokines have also been detected in the umbilical plasma, indicating an active role of these molecules in fetal development and metabolism [17]. To date, it is well known that adiponectin and leptin are the major adipokines involved in the regulation of insulin sensitivity in pregnancy, while the involvement of the other adipokines is still debated [18]. Recent studies demonstrated that some adipokines such as resistin, visfatin and tumor necrosis factor α (TNF-α) are dysregulated in GDM and contribute to metabolic complications typical of this pregnancy disorder [19, 20]. Table 1 summarizes data about adipokines in normal and GDM pregnancy.

**Table 1:** Tissue expression and maternal circulating levels of the main adipokines during normal pregnancy and gestational diabetes mellitus

Adipokine	Normal pregnancy levels	GDM pregnancy levels	Expression	Function	References
Leptin	Levels two to three times higher than in non-pregnant women; the peak occurs around the 28th week of gestation	Further increase ↑↑	Maternal adipose tissue Fetal adipose tissue Placenta	Promotion of fetal growth through greater placental lipolysis and transport of transplacental macronutrients Increased availability of fuel	[9, 22, 26, 30, 35]
Adiponectin	Progressively reduced levels	Further reduction ↓↓	Maternal adipose tissue Placenta, primarly in syncytiotrophoblast	Increased insulin-sensitivity Anti-inflammatory activity	[16, 48-50, 57, 64]
TNF-α	Higher levels than non-pregnant women, particularly in third trimester	Further increase ↑↑	Maternal adipose tissue Placenta	Aggravation of insulin resistance Regulation of placental development	[64, 76, 80, 81, 82, 83]
Resistin	Higher levels than non-pregnant women, particularly at the end of pregnancy	Further increase ↑↑	Maternal adipose tissue	Prevention of neonatal hypoglycemia Increased hepatic glucose production	[85-89]
Visfatin	Higher levels than non-pregnant women; the peak occurs between the 19th and 26th week of gestation	Further increase ↑↑	Maternal adipose tissue	Increased insulin-sensitivity Anti-inflammatory activity	[92-95]

The biological functions of the adipokines are also reported

### Leptin

Leptin is a 16 kDa peptide that circulates in serum as a free peptide or as a complex with α2-macroglobulin [21]. Leptin is mainly synthesized and released by the white adipose tissue (WAT), but is also produced by the gastrointestinal system, skeletal muscle, breast, ovary, placenta, pituitary gland, lymphoid tissue, mesenchymal stem cells and bone [9, 22]. Brown adipose tissue (BAT) was also found to be a source of leptin [23]. Leptin levels (ranging between 1 and 15 ng/mL) directly reflect the amount of energy stored in AT and are proportional to fat mass [24]. During pregnancy, a significant amount of leptin is produced by placental tissues and is secreted into fetal–maternal circulation; furthermore, leptin is present both in the amniotic fluid and in umbilical cord blood, although at lower levels than in maternal blood [25, 26].

Functionally, leptin is involved in many biological processes including regulation of appetite, energy homeostasis, insulin sensitivity, inflammation, immune response and angiogenesis. Regarding the reproductive system, leptin stimulates the secretion of the gonadotropin-releasing hormone (GnRH), the follicle-stimulating hormone (FSH) and the luteinizing hormone (LH) supporting fertility [18]. Leptin participates to the maintenance of energy homeostasis increasing both secretion and sensitivity to insulin, thus influencing glycogen synthesis and fatty acid metabolism [27]. Leptin also suppresses the synthesis and release of the neuropeptide oressigenous Y [24]. In obesity and related disorders such as T2DM, leptin levels are dramatically reduced; in leptin knowdown animal model, the deletion of the leptin gene and/or its receptor determines constant and continuous hunger, hyperphagia and early onset of severe obesity [28]. The above-mentioned biological functions of leptin are mediated by its interaction with its own receptor, expressed throughout the body, known as Ob-Re receptor [29]. About pregnancy, leptin is involved in the regulation of multiple aspects of maternal metabolic homeostasis such as the placentation process, the maternal–fetal exchanges, and the regular growth of the fetus. From the early stages of pregnancy, maternal concentrations of leptin increase to levels two or three times higher than those found in non-pregnant conditions, revealing an important role of this adipokine during gestation [8, 26]. The peak of leptin concentrations occurs at the end of the second or at the beginning of the third trimester; then, its levels remain elevated for the rest of the gestation, while drastically decreasing after the birth [30]. Several studies have shown that placental tissues, but not the maternal AT, contributes in defining this increase in leptin concentrations [30]. According to these data, Kinalski et al. showed a noticeable decrease in leptin after birth, indicating that the placenta is the main sources of elevated circulating leptin levels in pregnancy and that the expression of leptin in the placenta is independently regulated from that produced by AT [31]. In addition, the fetus itself contributes to the production of leptin starting from the second trimester, although with a lesser extent than the placenta [32].

Functionally, the increase of leptin in maternal blood during the second and third trimester of pregnancy is not associated with a decreased food intake or an increased metabolic activity [33]. Interestingly, it has been shown that leptin induces the production of chorionic gonadotropin in the trophoblast cells, thus regulating placental growth, improving mitogenesis and stimulating the uptake of amino acids [34, 35].

**Fig. 2:**
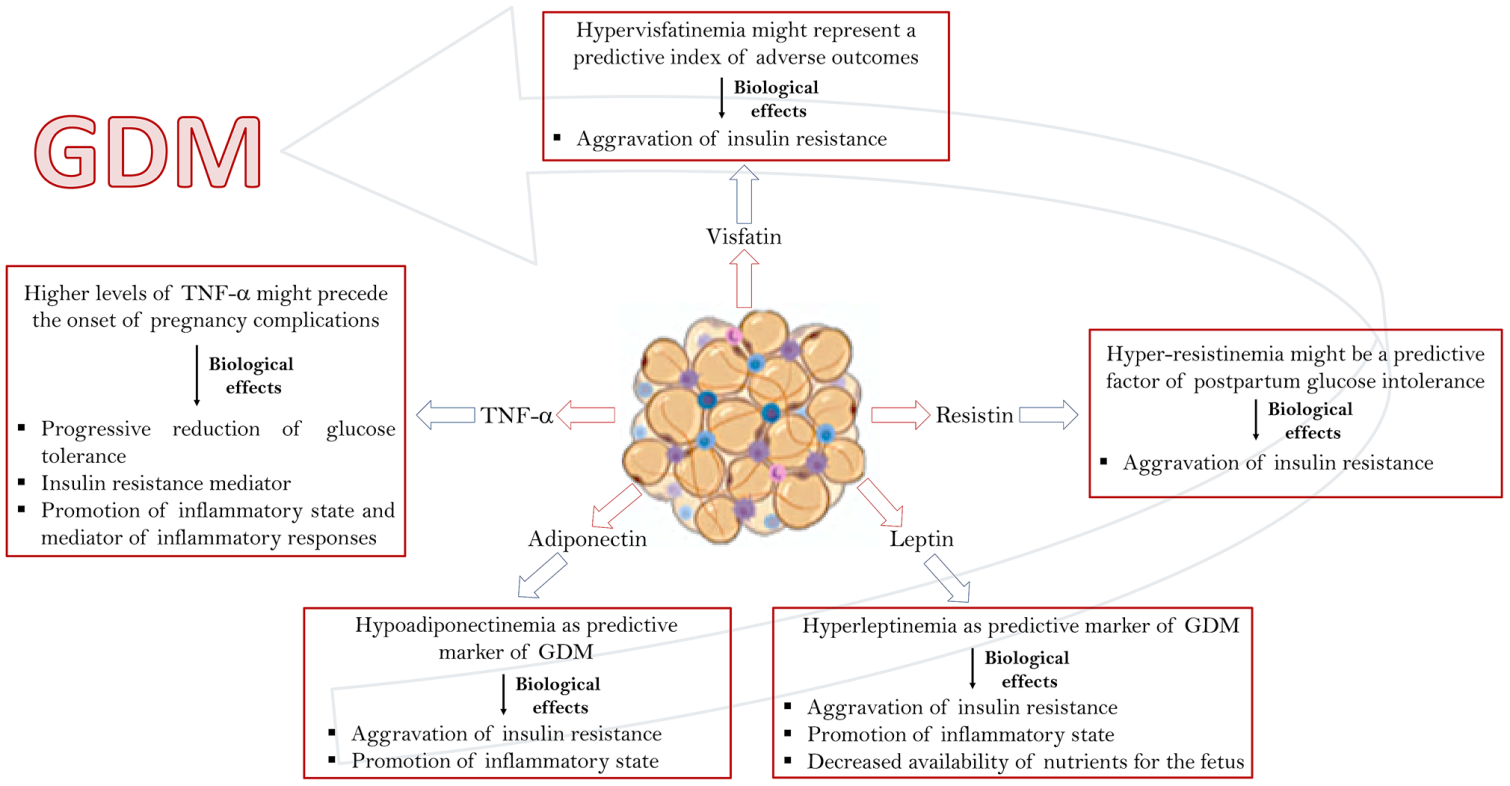
Role of the main adipokines involved in GDM. Leptin, adiponectin, TNF-α, resistin and visfatin are involved in the regulation of various aspects of maternal metabolism during normal and GDM-complicated pregnancy. Schematic representation of the altered levels of the above-mentioned adipokines together with their biological effects in GDM

When obesity occurs during the pregnancy, a resistance to leptin is observed with a decreased availability of nutrients for the fetus. This mechanism could explain some metabolic disorders at fetal level and the decrease of fetal growth that is observed in pregnancies complicated by obesity [36]. It has also been shown that leptin plays a role in the regulation of angiogenesis and motility of smooth muscle cells, indeed, in endothelial cells of the umbilical vein, leptin induces the phosphorylation of the vascular endothelial growth factor receptor 2 (VEGFR 2), a fundamental process for adequate fetal vascularization [37]. Leptin plays a pro-inflammatory action influencing the pathogenesis of various pregnancy diseases including GDM. Most of the literature data have associated hyperleptinemia with the development and progression of GDM, while only few studies have shown reduced or unchanged levels of this protein in affected women [38]. Differences in leptin levels between women with GDM and healthy ones could be due to a different expression of leptin receptors between the two groups. Challier et al. found an increase in soluble leptin receptor expression in the placenta of women with GDM, contrary to transmembrane protein receptor levels [2]. The increase in the soluble receptor determines a greater reuptake of leptin, limiting its availability for the transmembrane receptor [39]. This could affect the release of placental leptin and lead to a decrease of protein levels in GDM patients [39]. In contrast to these results, other studies have reported a decrease in soluble leptin receptor levels in GDM subjects [39, 40]. Recently, Fatima et al. detected a five times higher leptin levels in women with GDM than healthy controls and observed a positive correlation with fasting blood glucose, insulin resistance, and fetal weight [41]. Consistently, Xiao et al. showed an increase of leptin concentration in women with GDM compared to healthy ones [42]. It has been demonstrated that hyperleptinemia is more frequent in women with early onset of GDM compared to women with later onset of the disease and it is associated with a decrease in adiponectin/leptin ratio [43, 44]. Altogether, these studies showing leptin levels significantly increased in GDM women compared to control population suggest plausible role of leptin as a predictor factor for the onset of GDM [45, 46]. On the other hand, Thagaard et al. showed an inverse relation between leptin and GDM in severely obese women, claiming that the protein could not help in predicting the onset of the disease [39]. However, the presence of obesity may represent a confounding factor in defining the leptin as an early marker of GDM. Figure 2 summarizes the effects of leptin in GDM.

### Adiponectin

Cloned in 1990, adiponectin is an adipokine produced by AT and abundantly secreted in serum (ranging between 5 and 30 µg/mL), representing 0.01% of total serum proteins [48]. Adiponectin stimulates glucose consumption, inhibits lipolysis, and reduces hepatic glucose production with an important insulin-sensitizing function [49]. Altogether these functions result in anti-diabetic, anti-inflammatory and anti-aterogenic effects [50]. Circulating levels of adiponectin are inversely associated with glycemia, insulin sensitivity and inflammation and is largely recognized as a marker for obesity and metabolic-related diseases [51]. The adiponectin gene, located on chromosome 3q27, encodes a protein of 244 amino acids that multimerizes to form complexes at low (LMW), medium (MMW) and high molecular weight (HMW); the oligomers assemble through the formation of disulfide bridges and the hydroxylation of proline and lysine [52]. Adiponectin performs its biological functions through two transmembrane G-protein-coupled receptors: AdipoR1 and AdipoR2 [53]. Adiponectin receptors are widely expressed in many tissues with distinct expression patterns and different affinity for adiponectin oligomers [53]. McDonald et al. demonstrated that AdipoR1 and AdipoR2 are abundantly expressed also in human cytotrophoblast cells from term placentas [54].

In addition to the above receptors, adiponectin binds a third receptor, T-cadherin, a glycosylphosphatidylinositol-anchored extracellular protein lacking the intracellular domain with high affinity for HMW and MMW oligomers [55].

During pregnancy, adiponectin plays an important physiological role in maternal, fetal, and placental metabolism [56]. Generally, before the second trimester of pregnancy, there is an increase in circulating levels of adiponectin which progressively decrease with the advance of gestation [57]. The HMW oligomers are the most abundant circulating isoform in both pregnant and non-pregnant women [58]. Several studies reported that adiponectin is produced in abundance not only by AT, which for long time has been considered as the only source of this protein, but also by the placenta; the placental expression levels of adiponectin and its receptors are differently modulated by other cytokines, such as TNF-α, interferon-gamma (IFN-γ), interleukin 6 (IL-6) and leptin, that are crucial in the development of GDM [59, 60]. Contrary to these results, few studies did not detect adiponectin in placental tissues [58]. In addition to placenta, the fetus also contributes to adiponectin production, in particular in the early stages of the development with a specific production of HMW and LMW multimers [61]. Unlike non-pregnant women, adiponectin levels during pregnancy are not related to BMI, although a negative relationship between first and second trimester levels of this protein and pre-gestational BMI has been observed [62, 63]. In pregnant women, as for non-pregnant ones, maternal adiponectin improves insulin sensitivity by stimulating glucose consumption in skeletal muscles and reducing liver glucose production [64]. Functionally, adiponectin promotes the differentiation of the trophoblast cells, indicating that this adipokine could play a role in the formation of syncytiotrophoblast [65]. Contrary to the insulin sensitizing effects, maternal adiponectin has been shown to attenuate the signaling of insulin in the placenta and to reduce the maternal–fetal transport of amino acids, causing a decrease in fetal growth when produced at high levels [66]. Literature data report that, unlike adults, neonatal adiponectin positively correlates with some anthropometric parameters [67]. In fact, cord blood adiponectin levels positively correlate with fetal birth weight in both normal and diabetic pregnancies [68]. At birth, cord blood adiponectin levels are 4- to 7-fold higher than those observed in mothers [69]. The precise role of fetal adiponectin remains in part to be clarified; data collected suggest that adiponectin plays an important role in fetal intrauterine development and growth during the early stages of life.

The role of adiponectin has been extensively investigated in many pregnancy-associated disorders such as GDM. To date, GDM has been associated with a state of hypoadiponectinemia with a particular, deficiency of HMW oligomers [16, 70]. Indeed, many studies have reported reduced levels of circulating adiponectin in women with GDM compared with healthy ones [64]. Recently, this significant reduction in adiponectin gene expression has also been shown in affected women compared to healthy controls, regardless of their BMI [71]. Interestingly, in GDM women, adiponectin concentrations remain at low levels even after delivery [46]. Several studies have shown that, in early pregnancy, adiponectin levels decrease in GDM women compared to healthy controls, indicating that its concentration could be a predictive factor for the development of the disease already in the first trimester of pregnancy [39, 72]. Consistently, Lain et al. have shown that women with low adiponectin concentrations during the first trimester of pregnancy are 10 times more likely to develop GDM, while Williams et al. reported a 4.6-fold increase in the risk of developing GDM in women with low adiponectin concentrations [2]. Low adiponectin levels represent a predictive factor of GDM even in women without the known risk factors [73]. The molecular mechanism for adiponectin down-regulation is not clear but it is known that GDM is characterized by an amplification of the inflammatory state during pregnancy; this causes an increase in the circulating levels of many inflammatory cytokines including TNF-α and IL-6 which are negative regulators of the adiponectin expression [73]. It is to notice that fetuses of mothers with GMD show significantly lower adiponectin levels than those of healthy women and that the concentration of this adipokine remains low even during the growth of children [74]. The hypothesis is that low levels of this adipokine may further aggravate the insulin resistance that characterizes GMD. Considering that hyperinsulinemia during GDM may cause a further decrease in plasma levels of this adipokine, improving adiponectin levels in pregnant women could help improving insulin sensitivity and perinatal outcomes. Figure 2 summarizes the main roles of adiponectin in GDM.

#### TNF-α

TNF-α is a pro-inflammatory cytokine mainly produced by AT, monocytes and macrophages representing a key regulator of the immune system and inflammation [76]. It is well documented that TNF-α has a fundamental role in the development of insulin resistance and the pathogenesis of T2DM [77]. TNF-α inhibits the phosphorylation of insulin receptor and its substrate, insulin receptor substrate-1, thus, promoting insulin resistance. In addition, the expression of the glucose transporter type 4 (GLUT 4) is reduced by the activity of TNF-α in various tissues [64]. Treatment with TNF-α is known to decrease adiponectin expression in human adipocytes inactivating its insulin-sensitizing effects [78]. Thus, it is clear that elevated TNF-α levels during gestation may contribute to the reduced insulin sensitivity observed in pregnancy. TNF-α secretion increases during pregnancy in both normal and GDM women, particularly during the third trimester [64]. Additionally, a significant increase in TNF-α levels is reported in GDM mothers compared to healthy ones [79, 80]. Murthy et al. have shown that GDM women with elevated TNF-α levels are more likely to develop complications such as preeclampsia [2]. However, Rueangdetnarong et al. reported an increase of TNF-α levels in the maternal serum of GDM patients but not in cord blood, indicating that the placenta could be a barrier for pro-inflammatory cytokines [79]. According to Kirwan et al., the insulin-resistance and reduced glucose tolerance associated with GDM are precisely due to increased TNF-α levels rather than to direct activity of placental hormones [81]. Regarding the source of TNF-α secretion during pregnancy, the placenta represents the main source of TNF-α, with a peak of production in late gestation [82]. It seems that the increase in TNF-α levels during late phases of pregnancy is mainly due to placental activity rather than to secretion by other tissues [64]. After delivery, TNF-α levels decrease rapidly, supporting the hypothesis that its increase in pregnancy is due to placental secretion [83]. It seems clear that alterations in TNF-α levels could be involved in the pathogenesis of GDM, but the role of this adipokine is far from being elucidated. Figure 2 summarizes the main roles of TNF-α in GDM.

### Resistin

Resistin is a pro-inflammatory adipokine mainly produced by AT [85, 86]. This adipokine is involved in the regulation of insulin sensitivity and is known to be associated with obesity and insulin resistance in T2DM interfering with insulin function, affecting glycogen metabolism and decreasing glucose uptake in skeletal muscle [87]. The role of resistin in the development of insulin resistance associated with GDM remains unclear. During a physiological pregnancy, resistin level increases, particularly around the third trimester and probably contributes to the decrease of insulin sensitivity [86]. Some studies report a significant increase in the level of plasma and placental resistin in GDM patients compared to healthy ones [88]. A recent study, reported that between 11 and 13 weeks of pregnancy, the level of plasma resistin is increased in parturients who subsequently developed GDM, indicating that the hyper-resistinemia could precede the onset of the disease [20]. In addition, serum levels of resistin could be a predictor of postpartum glucose intolerance since the protein levels are higher in patients with GDM and postpartum glucose intolerance compared to patients with only GDM [89]. In contrast, other studies reported that there is no difference in circulating resistin levels between GDM patients and healthy controls and in the levels of umbilical resistin in children of mothers with GDM compared to children born from normal pregnancies [45, 91]. Lobo et al. have shown that there is no difference in resistin levels between women with early onset of GDM, women with standard onset of GDM and healthy controls [2]. In conclusion, the available data indicate that resistin in some way can influence insulin resistance during pregnancy but, most likely, it has a secondary role in the pathogenesis of GDM. Figure 2 summarizes the main roles of resistin in GDM.

### Visfatin

Visfatin is an adipokine highly expressed by the visceral adipose tissue (VAT). It has insulin-like effects and reduces the hepatic glucose levels by promoting glucose uptake in adipocytes and myocytes [92]. Literature data report that pregnant women have higher visfatin levels than non-pregnant women [93] with a peak between 19 and 26 weeks of gestation [94]. Regarding the role of visfatin in the pathogenesis of GDM, the results of literature studies are very heterogeneous. Lu et al. reported an increase in serum levels of maternal and umbilical cord visfatin in women with GDM compared to healthy controls [2]. They also showed that women with GDM with high levels of visfatin were more likely to have adverse outcomes, showing that visfatin could be a predictive index of the onset of adverse outcomes [95]. According to Souvannavong-Vilivong et al., the increase in serum visfatin levels found in women with GDM could be a compensatory mechanism to improve impaired insulin function [20]. Liang et al. revealed a positive correlation between perinatal levels of visfatin, weight gain and BMI in women with GDM [44]. In a recent study, an increase in the levels of visfatin was reported several weeks before the onset of GDM, indicating that this adipokine could be a potential biomarker for predicting the onset of the disorder [90]. On the contrary, Tsiotra et al. showed that circulating visfatin was significantly lower in GDM patients than in healthy controls although there were no differences in mRNA expression in AT and in the placenta [95]. In contrast to these results, in other studies, no difference in visfatin levels was found between mothers with GDM and healthy mothers [96, 97]. A recent meta-analysis analyzing the relationship between visfatin and GDM revealed that this adipokine is associated with GDM through maternal obesity, which represents one of the main risk factors for the onset of the disease [98]. The heterogeneity of these results do not allow elucidating the role that altered levels of visfatin play in GDM. Figure 2 summarizes the major roles of visfatin in GDM.

## Conclusions

GDM is a glucose intolerance pregnancy disorder associated with a higher risk of several short- and/or long‐term health problems for both the mother and offspring. Although the pathophysiology of GDM has not yet been fully elucidated, the available evidence suggests that the amount of AT before and/or during pregnancy influences insulin resistance associated with GDM-complicated pregnancies. Consequently, dysregulation in adipokine expression seems to be an essential factor that define and regulate insulin resistance and GDM. Adiponectin and leptin appear to be the main adipokines involved in the pathogenesis of GDM potentially representing markers and/or predictor factors of early GDM. Further studies are needed to clarify whether the dysregulation of the other described adipokines contributes directly to the pathophysiology of GDM.

## Data Availability

Not applicable.

## References

[1] Hauguel de Mouzon S, Lassance L (2015). Endocrine and metabolic adaptations to pregnancy; impact of obesity. Horm Mol Biol Clin Investig.

[2] Lowe WL, Karban J (2014). Genetics, genomics and metabolomics: new insights into maternal metabolism during pregnancy. Diabet Med.

[3] Catalano PM, Tyzbir ED, Roman NM, Amini SB, Sims EA (1991). Longitudinal changes in insulin release and insulin resistance in nonobese pregnant women. Am J Obstet Gynecol.

[4] Landon MB, Gabbe SG (2011). Gestational diabetes mellitus. Obstet Gynecol.

[5] Chen P, Wang S, Ji J, Ge A, Chen C, Zhu Y, Xie N, Wang Y (2015). Risk factors and management of gestational diabetes. Cell Biochem Biophys.

[6] Petry CJ (2010). Gestational diabetes: risk factors and recent advances in its genetics and treatment. Br J Nutr.

[7] Khan R, Ali K, Khan Z (2013). Socio-demographic risk factors of gestational diabetes mellitus. Pak J Med Sci.

[8] Plows JF, Stanley JL, Baker PN, Reynolds CM, Vickers MH (2018). The pathophysiology of gestational diabetes mellitus. Int J Mol Sci.

[9] Coelho M, Oliveira T, Fernandes R (2013). Biochemistry of adipose tissue: an endocrine organ. Arch Med Sci.

[10] Svensson H, Wetterling L, Bosaeus M, Odén B, Odén A, Jennische E, Edén S, Holmäng A, Lönn M (2016). Body fat mass and the proportion of very large adipocytes in pregnant women are associated with gestational insulin resistance. Int J Obes (Lond).

[11] Lappas M (2014). Effect of pre-existing maternal obesity, gestational diabetes and adipokines on the expression of genes involved in lipid metabolism in adipose tissue. Metabolism.

[12] Jayabalan N, Nair S, Nuzhat Z, Rice GE, Zuñiga FA, Sobrevia L, Leiva A, Sanhueza C, Gutiérrez JA, Lappas M, Freeman DJ, Salomon C (2017). Cross talk between adipose tissue and placenta in obese and gestational diabetes mellitus pregnancies via exosomes. Front Endocrinol (Lausanne).

[13] Poulos SP, Hausman DB, Hausman GJ (2010). Development and endocrine functions of adipose tissue. Mol Cell Endocrinol.

[14] Dutheil F, Gordon BA, Naughton G, Crendal E, Courteix D, Chaplais E, Thivel D, Lac G, Benson AC (2018). Cardiovascular risk of adipokines: a review. J Int Med Res.

[15] Poniedziałek-Czajkowska E, Mierzyński R, Dłuski D, Leszczyńska-Gorzelak B (2019). Adipokines and endothelium dysfunction markers in pregnant women with gestational hypertension. Int J Hypertens.

[16] Briana DD, Malamitsi-Puchner A (2009). Reviews: adipocytokines in normal and complicated pregnancies. Reprod Sci.

[17] Gutaj P, Sibiak R, Jankowski M, Awdi K, Bryl R, Mozdziak P, Kempisty B, Wender-Ozegowska E (2020). The role of the adipokines in the most common gestational complication. Int J Mol Sci.

[18] De Gennaro G, Palla G, Battini L, Simoncini T, Del Prato S, Bertolotto A, Bianchi C (2019). The role of adipokines in the pathogenesis of gestational diabetes mellitus. Gynecol Endocrinol.

[19] D’Ippolito S, Tersigni C, Scambia G, Di Simone N (2018). Adipokines, an adipose tissue and placental product with biological functions during pregnancy. Biofactors.

[20] Bawah AT, Seini MM, Abaka-Yawason A, Alidu H, Nanga S (2019). Leptin, resistin and visfatin as useful predictors of gestational diabetes mellitus. Lipids Health Dis.

[21] Ramos Lobo AM, Donato J (2017). The role of leptin in health and disease. Temperature (Austin).

[22] Reid IR, Baldock PA, Cornish J (2018). Effects of leptin on the skeleton. Endocr Rev.

[23] Zhang Y, Hufnagel C, Eiden S, Guo KY, Diaz PA, Leibel R, Schmidt I (2001). Mechanisms for LEPR-mediated regulation of leptin expression in brown and white adipocytes in rat pups. Physiol Genomics.

[24] Park HK, Ahima RS (2015). Physiology of leptin: energy homeostasis, neuroendocrine function and metabolism. Metabolism.

[25] Masuzaki H, Ogawa Y, Sagawa N, Hosoda K, Matsumoto T, Mise H, Nishimura H, Yoshimasa Y, Tanaka I, Mori T, Nakao K (1997). Nonadipose tissue production of leptin: leptin as a novel placenta derived hormone in humans. Nat Med.

[26] Valleau JC, Sullivan EL (2014). The impact of leptin on perinatal development and psychopathology. J Chem Neuroanat.

[27] Paz-Filho G, Mastronardi C, Wong ML, Licinio J (2012). Leptin therapy, insulin sensitivity, and glucose homeostasis. Indian J Endocrinol Metab.

[28] Zhou Y, Rui L (2013). Leptin signaling and leptin resistance. Front Med.

[29] Farr OM, Gavrieli A, Mantzoros CS (2015). Leptin applications in 2015: what have we learned about leptin and obesity. Curr Opin Endocrinol Diabetes Obes.

[30] Pérez Pérez A, Toro A, Vilariño García T, Maymó J, Guadix P, Dueñas JL, Fernández Sánchez M, Varone C, Sánchez Margalet V (2018). Leptin action in normal and pathological pregnancies. J Cell Mol Med.

[31] Kinalski M, Sledziewski A, Kowalska I, Telejko B, Kuźmicki M, Kretowski A, Majkowicz Młynarczyk A, Kinalska I (2004). Postpartum maternal plasma leptin levels and their relationship to gestational diabetes mellitus. Med Wieku Rozwoj.

[32] Lepercq J, Challier JC, Guerre Millo M, Cauzac M, Vidal H, Hauguel de Mouzon S (2001). Prenatal leptin production: evidence that fetal adipose tissue produces leptin. J Clin Endocrinol Metab.

[33] Tessier DR, Ferraro ZM, Gruslin A (2013). Role of leptin in pregnancy: consequences of maternal obesity. Placenta.

[34] Hauguel de Mouzon S, Lepercq J, Catalano P (2006). The known and unknown of leptin in pregnancy. Am J Obstet Gynecol.

[35] Henson MC, Castracane VD (2006). Leptin in pregnancy: an update. Biol Reprod.

[36] Brett KE, Ferraro ZM, Yockell Lelievre J, Gruslin A, Adamo KB (2014). Maternal–fetal nutrient transport in pregnancy pathologies: the role of the placenta. Int J Mol Sci.

[37] Garonna E, Botham KM, Birdsey GM, Randi AM, Gonzalez Perez RR, Wheeler Jones CP (2011). Vascular endothelial growth factor receptor-2 couples cyclo-oxygenase-2 with pro-angiogenic actions of leptin on human endothelial cells. PLoS ONE.

[38] Thagaard IN, Krebs L, Holm JC, Lange T, Larsen T, Christiansen M (2017). Adiponectin and leptin as first trimester markers for gestational diabetes mellitus: a cohort study. Clin Chem Lab Med.

[39] Challier J, Galtier M, Bintein T, Cortez A, Lepercq J, Hauguel-de Mouzon S (2003). Placental leptin receptor isoforms in normal and pathological pregnancies. Placenta.

[40] Sommer C, Gulseth HL, Jenum AK, Sletner L, Thorsby PM, Birkeland KI (2016). Soluble leptin receptor and risk of gestational diabetes in a multiethnic population: a prospective cohort study. J Clin Endocrinol Metab.

[41] Mosavat M, Omar SZ, Tan PC, Razif MFM, Sthaneshwar P (2018). Leptin and soluble leptin receptor in association with gestational diabetes: a prospective case–control study. Arch Gynecol Obstet.

[42] Fatima SS, Alam F, Chaudhry B, Khan TA (2017). Elevated levels of chemerin, leptin, and interleukin-18 in gestational diabetes mellitus. J Matern–Fetal Neonatal Med.

[43] Xiao WQ, He JR, Shen SY, Lu JH, Kuang YS, Wei XL, Qiu X (2020). Maternal circulating leptin profile during pregnancy and gestational diabetes mellitus. Diabetes Res Clin Pract.

[44] Lobo TF, Torloni MR, Mattar R, Nakamura MU, Alexandre SM, Daher S (2019). Adipokine levels in overweight women with early-onset gestational diabetes mellitus. J Endocrinol Investig.

[45] Manoharan B, Bobby Z, Dorairajan G, Vinayagam V, Packirisamy RM (2019). Adipokine levels and their association with insulin resistance and fetal outcomes among the newborns of Indian gestational diabetic mothers. Saudi Med J.

[46] Bozkurt L, Göbl CS, Baumgartner Parzer S, Luger A, Pacini G, Kautzky Willer A (2018). Adiponectin and leptin at early pregnancy: association to actual glucose disposal and risk for GDM—a prospective cohort study. Int J Endocrinol.

[47] Plowden TC, Zarek SM, Rafique S, Sjaarda LA, Schisterman EF, Silver RM, Yeung EH, Radin R, Hinkle SN, Galai N, Mumford SL (2020). Preconception leptin levels and pregnancy outcomes: a prospective cohort study. Obes Sci Pract.

[48] Achari AE, Jain SK (2017). Adiponectin, a therapeutic target for obesity, diabetes, and endothelial dysfunction. Int J Mol Sci.

[49] Orrù S, Nigro E, Mandola A, Alfieri A, Buono P, Daniele A, Mancini A, Imperlini E (2017). A functional interplay between IGF-1 and adiponectin. Int J Mol Sci.

[50] Frankenberg ADV, Reis AF, Gerchman F (2017). Relationship between adiponectin levels, the metabolic syndrome, and type 2 diabetes. Arch Endocrinol Metab.

[51] Ruan H, Dong LQ (2016). Adiponectin signaling and function in insulin target tissues. J Mol Cell Biol.

[52] Di Zazzo E, Polito R, Bartollino S, Nigro E, Porcile C, Bianco A, Daniele A, Moncharmont B (2019). Adiponectin as link factor between adipose tissue and cancer. Int J Mol Sci.

[53] Bianco A, Nigro E, Monaco ML, Matera MG, Scudiero O, Mazzarella G, Daniele A (2017). The burden of obesity in asthma and COPD: role of adiponectin. Pulm Pharmacol Ther.

[54] McDonald EA, Wolfe MW (2009). Adiponectin attenuation of endocrine function within human term trophoblast cells. Endocrinology.

[55] Hug C, Wang J, Ahmad NS, Bogan JS, Tsao TS, Lodish HF (2004). T-cadherin is a receptor for hexameric and high-molecular-weight forms of Acrp30/adiponectin. Proc Natl Acad Sci USA.

[56] Castro NP, Euclydes VV, Simões FA, Vaz-de-Lima LR, De Brito CA, Luzia LA, Devakumar D, Rondó PH (2017). The relationship between maternal plasma leptin and adiponectin concentrations and newborn adiposity. Nutrients.

[57] Fuglsang J, Skjaerbaek C, Frystyk J, Flyvbjerg A, Ovesen P (2006). A longitudinal study of serum adiponectin during normal pregnancy. BJOG.

[58] Arroyo-Jousse V, Jaramillo A, Castaño-Moreno E, Lépez M, Carrasco-Negüe K, Casanello P (2020). Adipokines underlie the early origins of obesity and associated metabolic comorbidities in the offspring of women with pregestational obesity. Biochim Biophys Acta Mol Basis Dis.

[59] Aye IL, Powell TL, Jansson T (2013). Review: adiponectin—the missing link between maternal adiposity, placental transport and fetal growth. Placenta.

[60] Chen J, Tan B, Karteris E, Zervou S, Digby J, Hillhouse EW, Vatish M, Randeva HS (2006). Secretion of adiponectin by human placenta: differential modulation of adiponectin and its receptors by cytokines. Diabetologia.

[61] Pinar H, Basu S, Hotmire K, Laffineuse L, Presley L, Carpenter M, Catalano PM, Hauguel de Mouzon S (2008). High molecular mass multimer complexes and vascular expression contribute to high adiponectin in the fetus. J Clin Endocrinol Metab.

[62] Ritterath C, Rad NT, Siegmund T, Heinze T, Siebert G, Buhling KJ (2010). Adiponectin during pregnancy: correlation with fat metabolism, but not with carbohydrate metabolism. Arch Gynecol Obstet.

[63] Mazaki Tovi S, Kanety H, Pariente C, Hemi R, Wiser A, Schiff E, Sivan E (2007). Maternal serum adiponectin levels during human pregnancy. J Perinatol.

[64] Świrska J, Zwolak A, Dudzińska M, Matyjaszek-Matuszek B, Paszkowski T (2018). Gestational diabetes mellitus—literature review on selected cytokines and hormones of confirmed or possible role in its pathogenesis. Ginekol Pol.

[65] Benaitreau D, Dos Santos E, Leneveu MC, De Mazancourt P, Pecquery R, Dieudonné MN (2010). Adiponectin promotes syncytialisation of BeWo cell line and primary trophoblast cells. Reprod Biol Endocrinol.

[66] Jones HN, Jansson T, Powell TL (2010). Full-length adiponectin attenuates insulin signaling and inhibits insulin-stimulated amino acid transport in human primary trophoblast cells. Diabetes.

[67] Tsai PJ, Yu CH, Hsu SP, Lee YH, Chiou CH, Hsu YW, Ho SC, Chu CH (2004). Cord plasma concentrations of adiponectin and leptin in healthy term neonates: positive correlation with birthweight and neonatal adiposity. Clin Endocrinol (Oxf).

[68] Aramesh MR, Dehdashtian M, Malekian A, ShahAli S, Shojaei K (2017). Relation between fetal anthropometric parameters and cord blood adiponectin and high-sensitivity C-reactive protein in gestational diabetes mellitus. Arch Endocrinol Metab.

[69] Kotani Y, Yokota I, Kitamura S, Matsuda J, Naito E, Kuroda Y (2004). Plasma adiponectin levels in newborns are higher than those in adults and positively correlated with birth weight. Clin Endocrinol (Oxf).

[70] Retnakaran A, Retnakaran R (2012). Adiponectin in pregnancy: implications for health and disease. Curr Med Chem.

[71] Ott R, Stupin JH, Melchior K, Schellong K, Ziska T, Dudenhausen JW, Henrich W, Rancourt RC, Plagemann A (2018). Alterations of adiponectin gene expression and DNA methylation in adipose tissues and blood cells are associated with gestational diabetes and neonatal outcome. Clin Epigenet.

[72] Madhu SV, Bhardwaj S, Jhamb R, Srivastava H, Sharma S, Raizada N (2019). Prediction of gestational diabetes from first trimester serum adiponectin levels in Indian women. Indian J Endocrinol Metab.

[73] Williams MA, Qiu C, Muy Rivera M, Vadachkoria S, Song T, Luthy DA (2004). Plasma adiponectin concentrations in early pregnancy and subsequent risk of gestational diabetes mellitus. J Clin Endocrinol Metab.

[74] Xu J, Zhao YH, Chen YP, Yuan XL, Wang J, Zhu H, Lu CM (2014). Maternal circulating concentrations of tumor necrosis factor-alpha, leptin, and adiponectin in gestational diabetes mellitus: a systematic review and meta-analysis. Sci World J.

[75] Kampmann FB, Thuesen ACB, Hjort L, Bjerregaard AA, Chavarro JE, Frystyk J, Bjerre M, Tetens I, Olsen SF, Vaag AA, Damm P, Grunnet LG (2019). Increased leptin, decreased adiponectin and FGF21 concentrations in adolescent offspring of women with gestational diabetes. Eur J Endocrinol.

[76] Idriss HT, Naismith JH (2000). TNF alpha and the TNF receptor superfamily: structure–function relationship(s). Microsc Res Tech.

[77] Akash MSH, Rehman K, Liaqat A (2018). Tumor necrosis factor-alpha: role in development of insulin resistance and pathogenesis of type 2 diabetes mellitus. J Cell Biochem.

[78] Ouchi N, Walsh K (2007). Adiponectin as an anti-inflammatory factor. Clin Chim Acta.

[79] Rueangdetnarong H, Sekararithi R, Jaiwongkam T, Kumfu S, Chattipakorn N, Tongsong T, Jatavan P (2018). Comparisons of the oxidative stress biomarkers levels in gestational diabetes mellitus (GDM) and non-GDM among Thai population: cohort study. Endocr Connect.

[80] Melekoglu R, Ciftci O, Celik E, Yilmaz E, Bastemur AG (2019). Evaluation of second trimester amniotic fluid ADAMTS4, ADAMTS5, interleukin-6 and tumor necrosis factor-α levels in patients with gestational diabetes mellitus. J Obstet Gynaecol Res.

[81] Sudharshana Murthy KA, Bhandiwada A, Chandan SL, Gowda SL, Sindhusree G (2018). Evaluation of oxidative stress and proinflammatory cytokines in gestational diabetes mellitus and their correlation with pregnancy outcome. Indian J Endocrinol Metab.

[82] Kirwan JP, Hauguel De Mouzon S, Lepercq J, Challier JC, Huston-Presley L, Friedman JE, Kalhan SC, Catalano PM (2002). TNF-α is a predictor of insulin resistance in human pregnancy. Diabetes.

[83] Chen H, Yang Y, Hu X, Yelavarthi K, Fishback J, Hunt J (1991). Tumor necrosis factor alpha mRNA and protein are present in human placental and uterine cells at early and late stages of gestation. Am J Pathol.

[84] Uvena J, Thomas A, Huston L, Highman T, Catalano PM (1999). Umbilical cord leptin and neonatal body composition. Am J Obstet Gynecol.

[85] Chen D, Dong M, Fang Q, He J, Wang Z, Yang X (2005). Alterations of serum resistin in normal pregnancy and pre-eclampsia. Clin Sci.

[86] Yura S, Sagawa N, Itoh H, Kakui K, Nuamah MA, Korita D, Takemura M, Fujii S (2003). Resistin is expressed in the human placenta. J Clin Endocrinol Metab.

[87] Siddiqui K, George TP (2017). Resistin role in the development of gestational diabetes mellitus. Biomark Med.

[88] Siddiqui K, George TP, Nawaz SS, Shehata N, El-Sayed AA, Khanam L (2018). Serum adipokines (adiponectin and resistin) correlation in developing gestational diabetes mellitus: pilot study. Gynecol Endocrinol.

[89] Karatas A, Tunçay Işikkent N, Ozlü T, Demirin H (2014). Relationship of maternal serum resistin and visfatin levels with gestational diabetes mellitus. Gynecol Endocrinol.

[90] Tsiotra PC, Halvatsiotis P, Patsouras K, Maratou E, Salamalekis G, Raptis SA, Dimitriadis G, Boutati E (2018). Circulating adipokines and mRNA expression in adipose tissue and the placenta in women with gestational diabetes mellitus. Peptides.

[91] Vitoratos N, Dimitrakaki A, Vlahos NF, Gregoriou O, Panoulis K, Christopoulos P, Creatsas G (2010). Maternal and umbilical resistin levels do not correlate with infant birth weight either in normal pregnancies and or in pregnancies complicated with gestational diabetes. J Matern–Fetal Neonatal Med.

[92] Adeghate E (2008). Visfatin: structure, function and relation to diabetes mellitus and other dysfunctions. Curr Med Chem.

[93] Morgan SA, Bringolf JB, Seidel ER (2008). Visfatin expression is elevated in normal human pregnancy. Peptides.

[94] Mazaki-Tovi S, Romero R, Kusanovic JP, Vaisbuch E, Erez O, Than NG, Chaiworapongsa T, Nhan- Chang CL, Pacora P, Gotsch F, Yeo L, Kim SK, Edwin SS, Hassan SS, Mittal P (2009). Maternal visfatin concentration in normal pregnancy. J Perinat Med.

[95] Lu D, Yang M, Yao Y, Xie Y (2019). A clinical research study on the respective relationships between visfatin and human fetuin A and pregnancy outcomes in gestational diabetes mellitus. Taiwan J Obstet Gynecol.

[96] Souvannavong-Vilivong X, Sitticharoon C, Klinjampa R, Keadkraichaiwat I, Sripong C, Chatree S, Sririwichitchai R, Lertbunnaphong T (2019). Placental expressions and serum levels of adiponectin, visfatin, and omentin in GDM. Acta Diabetol.

[97] Liang Z, Wu Y, Xu J, Fang Q, Chen D (2016). Correlations of serum visfatin and metabolisms of glucose and lipid in women with gestational diabetes mellitus. J Diabetes Investig.

[98] Görkem Ü, Küçükler FK, Toğrul C, Güngör T (2016). Are adipokines associated with gestational diabetes mellitus?. J Turk–Ger Gynecol Assoc.

[99] Zhang W, Zhao D, Meng Z, Wang H, Zhao K, Feng X, Li Y, Dun A, Jin X, Hou H (2018). Association between circulating visfatin and gestational diabetes mellitus: a systematic review and meta-analysis. Acta Diabetol.

